# Pancreaticogastrostomy versus Pancreaticojejunostomy and the Proposal of a New Postoperative Pancreatic Fistula Risk Score

**DOI:** 10.3390/jcm12196193

**Published:** 2023-09-25

**Authors:** Bogdan Mastalier, Victor Cauni, Constantin Tihon, Marius Septimiu Petrutescu, Bogdan Ghita, Valentin Popescu, Dan Andras, Ion Mircea Radu, Vasile Gabriel Vlasceanu, Marius Florian Floroiu, Cristian Draghici, Cristian Botezatu, Dragos Cretoiu, Valentin Nicolae Varlas, Angela Madalina Lazar

**Affiliations:** 1 Department of Surgery, Department of Functional Sciences, Faculty of General Medicine, Carol Davila University of Medicine and Pharmacy, 8 Eroii Sanitari Blvd., 050474 Bucharest, Romania; bogdanmastalier@yahoo.com (B.M.); popescu.vali.umf@gmail.com (V.P.); danandras69@gmail.com (D.A.); cristi_botezatu2001@yahoo.com (C.B.); angelalazar.2008@yahoo.com (A.M.L.); 2 General Surgery Clinic, Colentina Clinical Hospital, 020125 Bucharest, Romania; ioanatihon@yahoo.com (C.T.); mariuspetrutescu@yahoo.co.uk (M.S.P.); dr.bogdanghita@yahoo.com (B.G.); raduionmircea@gmail.com (I.M.R.); gabyvlasceanu@yahoo.ro (V.G.V.); 3 Urology Clinic, Colentina Clinical Hospital, 020125 Bucharest, Romania; victorcauni@yahoo.com; 4 Anaesthesia Intensive Care Unit, Colentina Clinical Hospital, 020125 Bucharest, Romania; floroium11@yahoo.it (M.F.F.); mihai.bogdan.draghici@gmail.com (C.D.); 5 Fetal Medicine Excellence Research Center, Alessandrescu-Rusescu National Institute for Mother and Child Health, 020395 Bucharest, Romania; 6 Department of Genetics, Carol Davila University of Medicine and Pharmacy, 8 Eroii Sanitari Blvd., 050474 Bucharest, Romania; 7 Department of Obstetrics and Gynaecology, Filantropia Clinical Hospital, 011171 Bucharest, Romania; 8 Department of Obstetrics and Gynaecology, “Carol Davila” University of Medicine and Pharmacy, 37 Dionisie Lupu St., 020021 Bucharest, Romania

**Keywords:** pancreaticoduodenectomy, pancreaticogastrostomy, pancreaticojejunostomy, pancreatic fistula, pancreatic fistula risk score, predictors, pancreatic fistula preventive strategies

## Abstract

Despite the substantial decrease in mortality rates following a pancreaticoduodenectomy to less than 5%, morbidity rates remain significant, reaching even 73%. Postoperative pancreatic fistula is one of the most frequent major complications and is significantly associated with other complications, including patient death. Currently, there is no consensus regarding the ideal type of pancreatic anastomosis, as the question of the choice between a pancreaticogastrostomy and pancreaticojejunostomy is still open. Furthermore, worldwide implementation of an ideal pancreatic fistula risk prediction score is missing. Our study found several significant predictive factors for the postoperative occurrence of fistulas, such as the soft consistency of the pancreas, non-dilated Wirsung duct, important intraoperative blood loss, other perioperative complications, preoperative patient hypoalbuminemia, and patient weight loss. Our study also revealed that for patients who exhibit fistula risk factors, pancreaticogastrostomy demonstrates a significantly lower pancreatic fistula rate than pancreaticojejunostomy. The occurrence of pancreatic fistulas has been significantly associated with the development of other postoperative major complications, and patient death. As the current pancreatic fistula risk scores proposed by various authors have not been consensually validated, we propose a simple, easy-to-use, and sensitive score for the risk prediction of postoperative pancreatic fistula occurrence based on important predictors from statistical analyses that have also been found to be significant by most of the reported studies. The new pancreatic fistula risk score proposed by us could be extremely useful for improved therapeutic management of cephalic pancreaticoduodenectomy patients.

## 1. Introduction

Recent advances in medicine, along with the refinement of surgical techniques and perioperative care, have resulted in a substantial decrease in mortality rates following cephalic pancreaticoduodenectomy to less than 5%. However, cephalic pancreaticoduodenectomy indicated for pancreatic head neoplasia (pancreatic duct adenocarcinoma being the most frequent type), as well as for ampullary, distal common bile duct, duodenal tumors, and rarely for pancreatic/duodenal trauma or chronic pancreatitis remains a very challenging procedure, with high morbidity rates in 40–50% and even up to 73% of cases [1,2,3,4,5,6,7]. Among the most frequent postoperative complications following cephalic pancreaticoduodenectomy are pancreatic fistulas, delayed gastric emptying, hemorrhage, intra-abdominal collections, and wound infection, with a significant percentage of patients (up to 74%) developing more than one postoperative complication [1,6]. Post-pancreaticoduodenectomy complications lead to important medical, economical, and human costs and represent a major cause of mortality [6,8,9].

It is remarkable that despite important developments in the surgical technique, pancreatic fistula rates remain very high, usually in the range of 5–30% but sometimes reaching up to 50% [1,2,3,4]. Such major variation in the frequency of postoperative pancreatic fistula reported by various authors is most likely due to initial differences in its definition and classification, with the first standardization brought by the International Study Group on Pancreatic Fistula in 2005 and its update in 2016 [1,2,4].

Given the high rates of postoperative pancreatic fistula, various types of anastomoses have been proposed in order to decrease the risk of pancreatic leakage and other postoperative complications, with more than 70 variations from the original classic Whipple operation; however, no consensus has been reached regarding the superiority of any of the techniques [2,10]. In this regard, there are major controversies regarding the choice of the pancreatic anastomosis type—pancreaticogastrostomy versus pancreaticojejunostomy [4]—and alternative attitudes regarding the pancreatic duct: ligation, occlusion with cyanoacrylate/hemostatic patch sealant, or even total pancreatectomy [11,12]. Also, no conclusions could be drawn on the utility of internal or external stents across the pancreaticoenteric anastomosis, the order of the suturing of the anastomoses, or regarding the differences between pylorus-preserving pancreaticoduodenectomy and the classic technique [13,14]. In fact, only for the pancreaticogastrostomy and pancreaticojejunostomy have a tremendous multitude of anastomotic technique subtypes been proposed, such as end-to-side duct-to-mucosa anastomosis and one-layer or two-layer anastomosis [4,15]; end-to-side invagination or dunking technique of pancreaticojejunostomy; end-to-end telescoping technique; the binding Peng’s technique of pancreaticojejunostomy; pylorus-preserving pancreaticoduodenectomy with gastric partition followed by end-to-side duct-to-mucosa or invagination technique; purse-string anastomosis; stented and unstented anastomoses; isolated Roux loop pancreaticojejunostomy; precolic or retrocolic positioning of the anastomosis; pancreaticogastrostomy or pancreaticojejunostomy with two transpancreatic sutures with buttresses; and mesh reinforcement of the pancreaticojejunostomy or omental wrapping of the anastomosis [1,2,3,4,8,10,13,16,17,18,19,20,21,22]. At the same time, in order to decrease the risk of hemorrhage and other complications, identify anatomic arterial anomalous origins/variations or arterial stenosis, and facilitate a radical/extended dissection, two main pancreatic head dissection approaches have been described: the posterior or artery-first (with multiple technical variants) and anterior or uncinate-first approaches. Other approaches have been proposed as well: inferior supracolic/anterior approach, inferior infracolic/mesenteric approach, combination of anterior and posterior approaches (hanging maneuver), right/medial uncinate approach, superior approach, left posterior approach, and a combination of multiple artery-first approaches [5,23,24,25,26]. Although many authors prefer an artery-first approach, there is no current consensus on which approach is the best [24,26,27].

The problem of the pancreatic stump and pancreatic anastomosis after cephalic pancreaticoduodenectomy is still regarded as the Achilles’ heel today because it represents one of the most challenging, difficult-to-achieve techniques in general surgery; the overall outcome of the surgical operation and patient prognosis depends on its success [18,20,22]. Pancreatic anastomosis is different from other anastomoses for multiple reasons, such as the following: pancreatic consistency is normally fragile and suturing is difficult, with high risk of lacerations and leakage; the small diameter of the pancreatic duct; the pancreas is fixed to the retroperitoneum; the pancreas’ proximity to important blood vessels, such as the superior mesenteric artery and portal vein, as well to various organs, such as the stomach and duodenum, which make achievement of the anastomosis more difficult; the hard consistency of the pancreas, as a result of tissue fibrosis, associated with a lower postoperative pancreatic fistula risk, as the hard consistency has a lower risk of laceration during suture; and fibrotic changes obstructing the ducts, leading to lower pancreatic secretion flow [27,28].

Although some authors prefer pancreaticogastrostomy over pancreaticojejunostomy, there is still no current consensus on the superiority of the pancreaticogastrostomy versus pancreaticojejunostomy or regarding any of the multiple proposed surgical techniques, as the reported results are inconsistent [1,2,4,6,29]. Therefore, in this context, the question “pancreaticogastrostomy or pancreaticojejunostomy?”, first presented by Kausch and Whipple, still remains open today [8,30]. In this regard, a few reported meta-analyses and randomized trials have found that pancreaticogastrostomy might be better as it was associated with a smaller rate of pancreatic fistulas in the conducted studies. However, the majority of the reported studies have not found a significant difference between the pancreaticogastrostomy and pancreaticojejunostomy procedures regarding the risk of pancreatic fistula occurrence [1,4,8,31].

Multiple advantages and disadvantages for both the pancreaticogastrostomy and pancreaticojejunostomy procedures have been communicated so far. A pancreaticogastrostomy is considered to be easier to perform (less technically demanding) than a pancreaticojejunostomy and its advantages include the following: the anatomic proximity, rich gastric vascularization, the gastric low pH preventing the activation of the pancreatic enzymes or inactivating them, absence of the enterokinase, and better postoperative control (endoscopy) with the possibility of gastric decompression and a putative lower risk of pancreatic fistula [2,8,30]. However, there are also disadvantages associated with such an anastomosis: difficult hemostasis of the gastric submucosa, a more difficult mobilization of the pancreas, increased risk of hemorrhage, postoperative delayed gastric emptying (due to gastroparesis and possible tensions on the anastomosis), and risk of pancreatic enzymes inactivation. According to some studies, although no consensus has been reached yet, exocrine pancreatic deficiency is more frequent after a pancreaticogastrostomy than after a pancreaticojejunostomy [1,2,3,6,29,30,31,32]. Also, there is a potential difference in the length of surgery between the pancreaticogastrostomy and pancreaticojejunostomy procedures. In this regard, a pancreaticojejunostomy appears to be faster, as more time is needed for a pancreaticogastrostomy: to create an anterior gastrotomy, posterior gastrotomy, and closure of the anterior gastrotomy, a longer mobilization of the pancreas is needed to bring it to the non-movable posterior gastric wall, while the jejunum is more easily movable. Instead, the advantages of performing a pancreaticojejunostomy are the mobility of the jejunum, good vascularization, and better preservation of the pancreatic exocrine function, while its disadvantages are mainly represented by the activation of the pancreatic enzymes by the enteric juice, leading to an increased risk of complex fistulas [19,28,33].

In 2005, the International Study Group on Pancreatic Fistula (ISGPF) first defined pancreatic fistula as being any volume of fluid output via an operatively placed drain, with amylase activity that is more than three times the upper normal serum level [34]. At that time, three clinical grades of pancreatic fistula were proposed: A, B, and C—a grading that has been used on over 320,000 patients in original studies over a period of ten years. However, in 2016, the ISGPF released an update, stating that grade A is no longer a true, clinically relevant fistula but a transient, asymptomatic, “biochemical fistula”; only if the drain must be maintained for more than 3 weeks is the fistula considered clinically relevant for the patient. Therefore, if no drains are placed in no-risk or low-risk patients, grade A is never recorded [34,35].

For improved prediction, earlier diagnosis, and more efficient therapy, multiple fistula prediction scores, based on pre- and/or intraoperative factors, have been designed up to now. One is the Callery score, also known as fistula risk score, which appears to associate the best predictive power from all the scores (however, not an ideal correlation); some other scores include the Wellner score, Roberts score, and Yamamoto score (with apparent no predictive significance when tested in a few studies) [36,37]. There are also fistula risk score calculators available online; however, despite the multitude of proposed scores, there is still no consensus on the best fistula score [35,38,39].

Facing such limitations, the aims of the current study were to establish which type of anastomosis, pancreaticogastrostomy or pancreaticojejunostomy, is better in terms of postoperative results and associated with a lower risk of pancreatic fistulas. Also, the study aimed to identify putative predictive factors for the occurrence of the postoperative pancreatic fistula, their effect on the pancreatic fistula’s degree of severity, and to elaborate an improved fistula score based on significant predictors that resulted from the statistical analysis. Such identification of pancreatic fistula predictive factors would be essential for a differentiated surgical approach of the cases according to the risk of fistula development, for an early fistula diagnosis and treatment, and better therapeutic management of the patients based on the fistula grade of severity.

## 2. Materials and Methods

**Figure 1:**
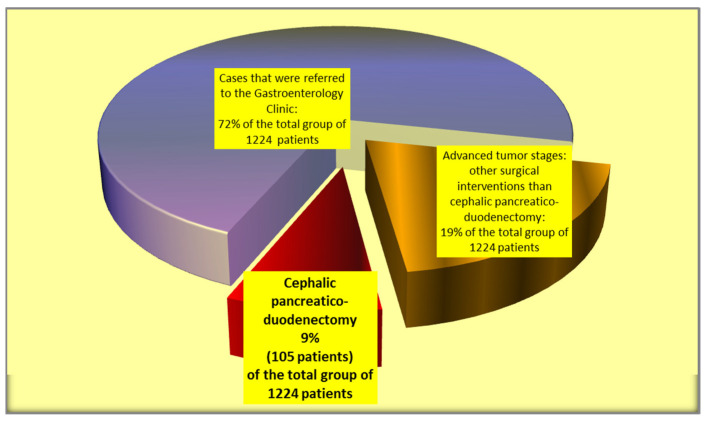
Selection of the cases included in the study group.

The current retrospective study was performed over a period of 12 years between 2010 and 2021. During the study period, 1224 patients were diagnosed and treated for pancreatic head and periampullary tumors in Colentina Clinical Hospital, Bucharest, Romania. From the reported group, 881 patients were diagnosed/treated in the Gastroenterology Clinic (endoscopic procedures) while only 343 patients were addressed to the Surgery Clinic (First and Second Surgical Clinics of Colentina Clinical Hospital), where pancreatic surgeries (cephalic pancreaticoduodenectomy, palliative surgical interventions, and various surgical digestive and biliary diversions) were performed. For the current study, we included only the patients (105 cases) where cephalic pancreaticoduodenectomy was performed (Figure 1). As one of the main aims of the study is to identify putative predictors for the occurrence of pancreatic fistulas after cephalic pancreaticoduodenectomy, we did not include the patients where total pancreatectomy (13 cases) or other types of surgical interventions were performed. During the study period, in the selected study group of 105 patients with cephalic pancreaticoduodenectomy, no cases of neoadjuvant therapy were recorded. All the cases included in the study were operated on by the same surgical team. For our retrospective study, given the ISGPF changes in the classification of the postoperative pancreatic fistula after cephalic pancreaticoduodenectomy, we used the ISGPF fistula definitions and grading from 2016. Therefore, all the cases of pancreatic fistula from the study represented clinically relevant postoperative pancreatic fistulas (grades B or C). We analyzed the types of surgical interventions achieved for this group of patients and their frequency, and the prognostic differences between various types of surgical interventions, especially between the pancreaticojejunostomy and pancreaticogastrostomy procedures. In order to identify putative pancreatic fistula predictors, elaborate the pancreatic fistula score, and compare pancreaticogastrostomy versus pancreaticojejunostomy in terms of therapeutic results and patient postoperative prognosis, a comprehensive statistical analysis on an extended array of variables was conducted, including patient-related factors, diagnostic procedures, tumor characteristics (localization, dimension, histopathologic type, tumor grading), vascular involvement features, tumor invasion into adjacent tissues, lymph node dissemination and distant metastasis, TNM tumor stage, the performed type of surgery, intraoperative and postoperative complications, and early and long-term results after surgery. Microsoft Office Excel 2010 (Microsoft Office Home and Student 2010, Microsoft Corporation One Microsoft Way Redmond, WA 98052 USA) and Epi Info (TM) version 3.5.1. software (Epi Info (TM) developed by Centers for Disease Control and Prevention (CDC) in Atlanta, Georgia (US), release date August 2008, available at https://www.cdc.gov/epiinfo/support/downloads/prevversions.html (accessed on 20 September 2023) were used for statistical analyses. The study was based on a descriptive statistical anaysis of data (median, mean values, minimum, maximum, range) and comparisons between groups using Student‘s *t*-test, chi-squared test, and Fisher’s exact test, as required. Only *p*-values that were less than 0.05 were considered to be significant throughout the statistical analysis. The study was conducted in accordance with the principles of the Declaration of Helsinki.

## 3. Results

Only 31% of the cases of pancreatic and periampullary tumors (105 patients included in the study) evaluated in Colentina Clinical Hospital were suitable for cephalic pancreaticoduodenectomy. Instead, a larger percentage of cases represented advanced tumor stages where only bile and digestive derivations or endoscopic bile drainage could be performed.

For the group of patients where cephalic pancreaticoduodenectomy was performed, the most frequent cases were pancreatic head tumors, especially adenocarcinomas.

**Figure 2:**
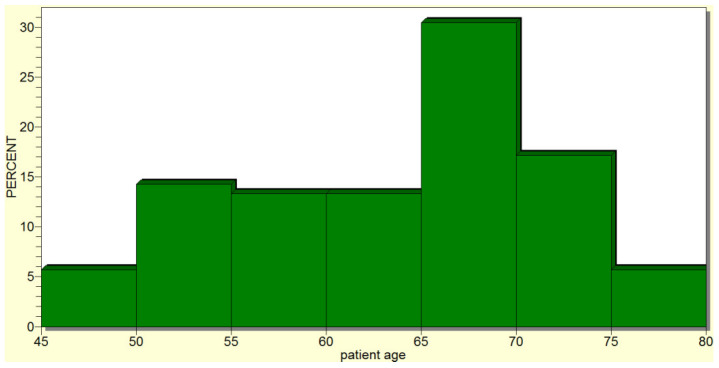
Cephalic pancreaticoduodenectomy was most frequently performed in older patients.

The mean age of the cephalic pancreaticoduodenectomy patients was 63.4190 ± 8.2039 years (median of 66 years), with the minimum age being 45 and the maximum being 79 years (Figure 2). Therefore, cephalic pancreaticoduodenectomy was usually performed in older patients, with the most frequently operated-on age group being that of 60–65 years.

For the studied group of patients, cephalic pancreaticoduodenectomy was performed more frequently for male patients, with the male-to-female ratio being 1.6/1. At patient admission, the most frequent complaints were jaundice, abdominal pain, weight loss, and fatigue.

Taking into account the complexity of the surgical technique—the significant number of major structures (blood vessels and organs) that can be invaded by the tumor or surgically injured—a detailed preoperative evaluation of the tumor’s existence, extension, invasion, or proximity to major structures was achieved via multiple diagnostic means, such as abdominopelvic CT, abdominopelvic echography, MRI, endoscopy/echoendoscopy, and ERCP.

**Figure 3:**
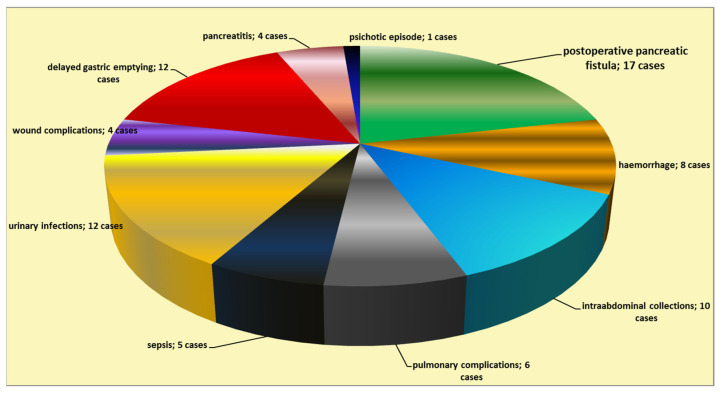
Types of postoperative complications following cephalic pancreaticoduodenectomy.

The postoperative mortality rate was 4.8% of the cases (five patients), while the morbidity rate was 32.4% (in 34 cases). The most frequent postoperative complications were the occurrence of pancreatic fistula (in 17 patients; 16.2% of the operated-on cases), delayed gastric emptying (in 12 patients), intra-abdominal collections (in 10 patients), hemorrhage (eight cases), and general complications (Figure 3). A grade C postoperative pancreatic fistula was found in 29.4% of the cases of fistula (five cases), while the remainder of the fistula cases were grade B (70.6% of the cases of fistula—12 patients).

The most frequent histopathologic diagnoses were pancreatic adenocarcinoma, ampullary adenocarcinoma, cholangiocarcinoma, and duodenal carcinoma (Table 1).

**Figure 4:**
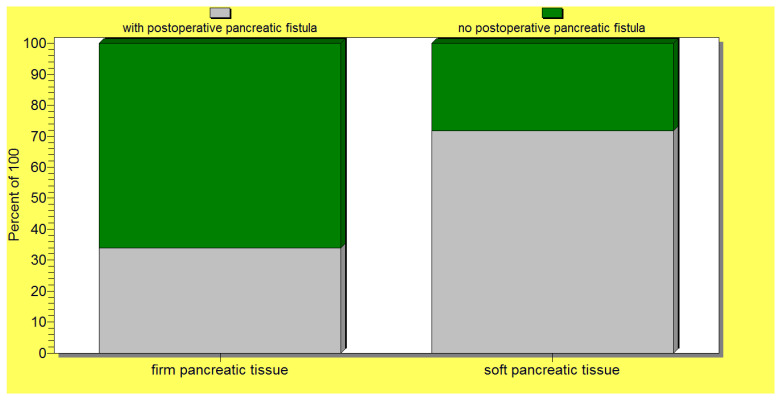
A significant association between a soft pancreatic texture and the occurrence of postoperative pancreatic fistulas.

**Figure 5:**
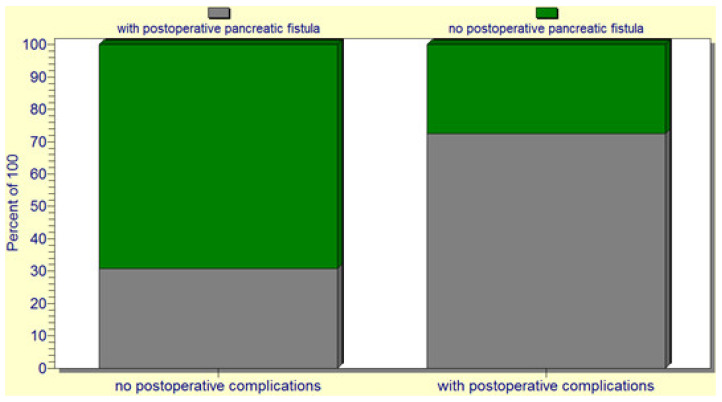
The pancreatic fistula leads to other important postoperative complications; at the same time, other postoperative complications increase the risk of pancreatic fistula.

The statistical analysis revealed a significant correlation (*p* = 0.02) between the occurrence of postoperative pancreatic fistulas and patient death. Several statistically significant predictors for the occurrence of pancreatic fistulas were found: preoperative patient weight loss and low levels of albumin (*p* = 0.0001); intraoperative hemorrhage (*p* = 0.0004); soft consistency of the pancreas (*p* = 0.007) (Figure 4); non-dilated (small or normal) pancreatic duct (*p* = 0.009); and the occurrence of other postoperative complications (*p* = 0.001). In this regard, a vicious feedback loop could be described: pancreatic fistula leads to other important postoperative complications; however, at the same time, other postoperative complications increase the risk of pancreatic fistulas (contributing role/trigger) (Figure 5). Also, a positive correlation between increased body mass index and soft consistency of the pancreas was found (*p* = 0.01).

Instead, several putative factors were not validated as significant predictors for the development of postoperative pancreatic fistulas (*p* > 0.05), such as intrapancreatic stenting (*p* = 0.19), patient characteristics (age, sex) (*p* = 0.32), patient comorbidities (such as cardiac comorbidities, history of diabetes mellitus (*p* = 0.21), and others), patient preoperative obesity (*p* = 0.05), smoking status (*p* = 0.3), pancreatic tumor dimension (maximal diameter) (*p* = 0.4), and tumor histopathologic type (*p* = 0.1).

**Figure 6:**
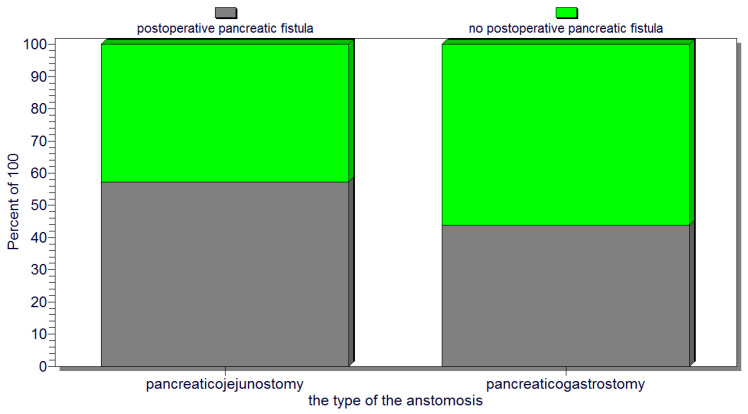
No significant association between the type of pancreatic anastomosis (pancreaticojejunostomy or pancreaticogastrostomy) and the occurrence of pancreatic fistulas.

Also, for the entire group of patients with cephalic pancreaticoduodenectomy, there was no statistically significant association between the type of pancreatic anastomosis, pancreaticojejunostomy (representing 44.8% of the cases—47 patients) versus pancreaticogastrostomy (performed in 55.2% of the cases—58 patients), and the occurrence of pancreatic fistulas (*p* = 0.2) (Figure 6). Also, no significant correlation could be found between the type of pancreatic anastomosis (pancreaticojejunostomy versus pancreaticogastrostomy) and postoperative patient death.

**Figure 7:**
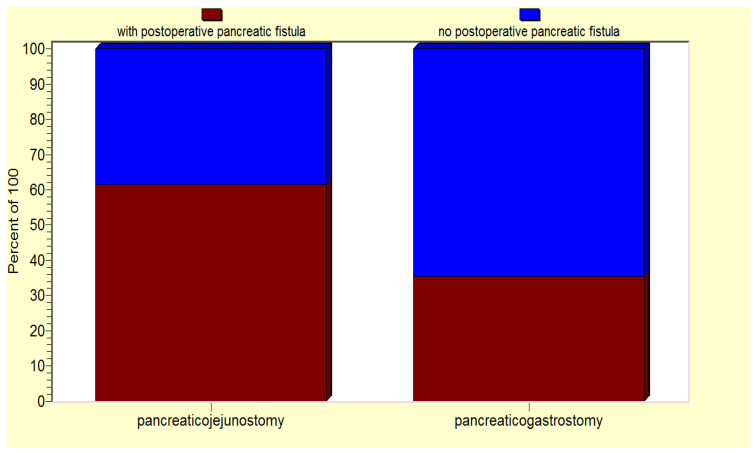
For the high-risk patient group, pancreaticogastrostomy is associated a lower risk of postoperative pancreatic fistula.

From the entire group, we selected a subgroup of 67 patients (63.8% of all the operated-on patients) characterized by a higher risk of pancreatic fistula development due to their associations of one/several risk factors, as revealed by the statistical analysis. Interestingly, for this subgroup of patients, there was a significant correlation between the performance of a pancreaticogastrostomy and a lower risk of postoperative pancreatic fistula (*p* < 0.05) (Figure 7).

**Figure 8:**
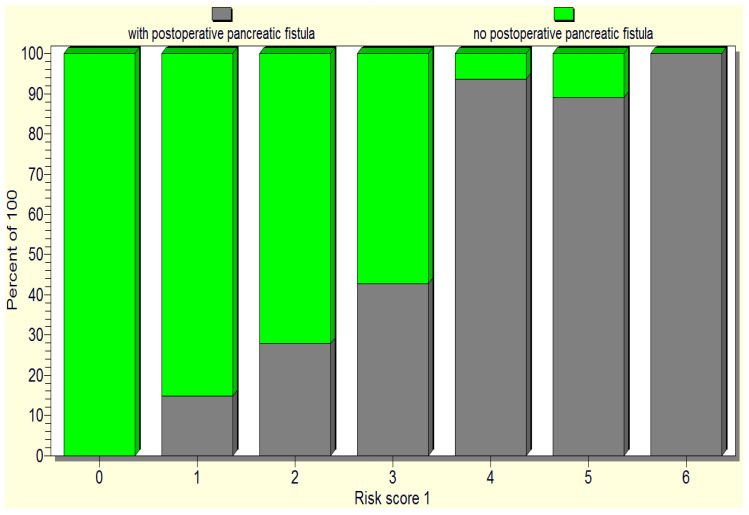
The repartition of the cases based on the initially proposed pancreatic fistula risk scale.

Considering the predictors of pancreatic fistula occurrence, as revealed by the statistical analysis (soft pancreas, non-dilated Wirsung duct, preoperative patient weight loss, hypoalbuminemia, intraoperative hemorrhage, and the occurrence of postoperative complications), we designed a pancreatic fistula risk score, where each risk factor received one point, with the scale range being 0 to 4 points: a minimum score of 0 points indicates no fistula risk; a score of 1–2 points is considered as a low risk of pancreatic fistula; 3 points denotes a medium risk of pancreatic fistula; 4–6 points indicates a high risk of pancreatic fistula (Figure 8).

When tested, the initial pancreatic fistula risk scale only proved a significant difference between high versus low risk of pancreatic fistula. Therefore, we reconsidered the score with only three categories and developed a new fistula score: fistula unlikely, 0 points; low risk, 1–3 points; high risk, 4–6 points (Table 2).

**Figure 9:**
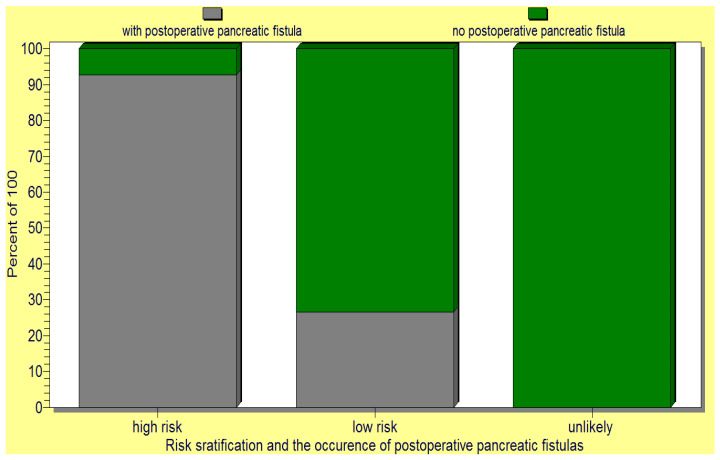
The new pancreatic fistula score proposed by us significantly correlated (*p* < 0.05) with the occurrence of postoperative pancreatic fistula.

**Figure 10:**
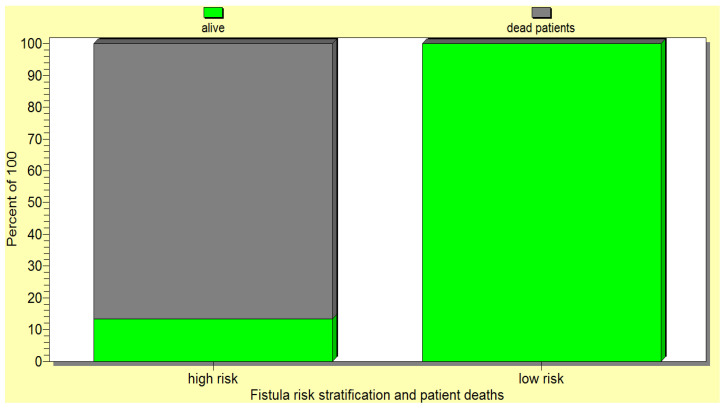
The new pancreatic fistula score proposed by us significantly correlated with the death of the operated-on patients.

The above pancreatic fistula score was validated by the statistical analysis as it significantly correlated (*p* < 0.05) with the occurrence of postoperative pancreatic fistulas as well as the death of operated-on patients (Figure 9 and Figure 10).

## 4. Discussion

Currently, there are still many controversies concerning cephalic pancreaticoduodenectomy: regarding the best pancreatic anastomosis (pancreaticogastrostomy versus pancreaticojejunostomy and their multiple sub-variants); about the use of pancreatic duct stents or not; between different surgical techniques—duct-to-mucosa, invagination, dunking, etc.; and regarding the optimal risk score to be used for the prediction of the postoperative pancreatic fistula [3]. Most of these controversies come from the limitations of the reported studies, meta-analyses, and randomized controlled studies, such as no real randomization (in many cases as the surgeon chooses intraoperatively, according to the local anatomy), the type of anastomosis that is best-suited for the case, the retrospective aspect of many studies, lack of data, confounders and other sources of bias, differences in fistula definition and grading over time, heterogeneity among the reported studies, and the small number of studies included in meta-analyses [4,6,8,14,16,28,36,40].

Therefore, there is no consensus regarding the choice of the ideal type of pancreatic anastomosis following cephalic pancreaticoduodenectomy, with conflicting/inconclusive results between the reported studies [21,41,42,43]. In this regard, some authors opt for the pancreaticojejunostomy while others prefer the pancreaticogastrostomy procedure, with comparable results, similar rates of clinically relevant pancreatic fistula, overall postoperative complications, and mortality between the two techniques, as reported by many studies [33,35]. While the pancreaticogastrostomy procedure was relatively recently proposed as an alternative, safer technique with many advantages, the pancreaticojejunostomy is still frequently performed nowadays, with multiple variants [18,28,44,45]. In this context, most authors consider the following question: “Which is safer: pancreaticogastrostomy or pancreaticojejunostomy?”. This question is still open, as the majority of studies have failed to prove the superiority of a particular technique. The remarkable expansion in the sub-variants of the pancreatic anastomosis technique, for the pancreaticojejunostomy as well the pancreaticogastrostomy [2,8], and the frequently small cohorts of patients included in the reported studies, impede a comparative analysis between the two major types and between the various sub-variants of the techniques. In this regard, the existing comparative subgroup analysis and meta-analysis on different techniques have not led to a clear conclusion regarding the best surgical technique [4]. However, knowledge of the type of pancreatic anastomosis that is associated with the lowest postoperative complications, such as the occurrence of pancreatic fistula, is crucial to improve the otherwise dismal prognosis of patients following cephalic pancreaticoduodenectomy.

In our study, when testing the entire group of patients, we could not find a significant association between the type of pancreatic anastomosis (pancreaticojejunostomy versus pancreaticogastrostomy) and the actual occurrence of postoperative pancreatic fistulas. Therefore, such a finding confirms why currently there is no unanimous recommendation on the execution of a certain type of pancreatic anastomosis, as the majority of authors report similar results (non-significant differences) between the two types [10,46]. However, in our study, when considering only the sub-group of patients with associated fistula risk factors, there was a significant correlation between the pancreaticogastrostomy procedure and a lower risk of postoperative pancreatic fistula when compared with the pancreaticojejunostomy procedure. Therefore, there were no significant differences in terms of postoperative pancreatic fistula occurrence between a pancreaticogastrostomy and pancreaticojejunostomy when the entire group of study was analyzed. Instead, we found that a pancreaticogastrostomy was associated with a significantly lower risk of pancreatic fistula compared with pancreaticojejunostomy only when the analysis was performed on the high-risk group of patients (the patients associated with one or several factors of risk for postoperative pancreatic fistula occurrence). Other authors have also reported a lower risk of pancreatic fistula following a pancreaticogastrostomy compared with a pancreaticojejunostomy, especially in the case of high-risk patients, although a pancreaticogastrostomy is usually associated with a higher risk of hemorrhage [43,47,48]. We consider such a finding of particular significance as it can be essential in the choice of the surgical approach and technique. In fact, many surgeons always perform a pancreaticogastrostomy when dealing with a soft pancreas or small pancreatic duct [8,17]. In this context, some authors have tried to explain the mechanism behind a smaller risk of pancreatic fistula following a pancreaticogastrostomy when compared with a pancreaticojejunostomy, with the putative explanation being the different healing capacity and vascularization of the gastric versus jejunal wall [4,6,37,47,49].

Similar to the reports of other authors [50,51,52,53,54,55], our study found an important postoperative morbidity rate of 32%, where the most frequent complications are as follows: pancreatic fistula (in 17 patients, representing 16.2% of the operated-on cases); delayed gastric emptying; intra-abdominal collections; hemorrhage; and general complications. The postoperative pancreatic fistula occurrence rate from our study falls as an average value in the wide range of fistula rates reported by other authors (5–30%, up to 73%, with large variability between studies) [1,2,3,4,5,6,7]. Post-pancreaticoduodenectomy hemorrhage refers to pancreatic hemorrhage, gastrointestinal bleeding, and intraperitoneal hemorrhage, although many studies do not specify the exact type of hemorrhage [4]; meanwhile, the mechanism behind postoperative delayed gastric emptying is not sufficiently understood [14,50].

It is important to stress that the occurrence of postoperative pancreatic fistula represents one of the most frequent complications, is a leading cause of morbidity (intra-abdominal collections/abscesses, peritonitis, pancreatic hemorrhage, infection of the surgical wound) and mortality after cephalic pancreaticoduodenectomy, and delays or prevents patient access to adjuvant therapy [43,46]. Our study has revealed a similar finding, with the postoperative pancreatic fistula being recorded in 16.2% of the operated-on cases and being the most frequent cause of postoperative morbidity along with delayed gastric emptying, intra-abdominal collections, hemorrhage, and general complications. In this regard, we found a significant association between the occurrence of pancreatic fistula and other postoperative complications: the pancreatic fistula can lead to other complications; at the same time, the occurrence of other postoperative complications increases the risk of postoperative fistula development (a vicious circle), as also described by other authors [40]. We also found a significant correlation between the occurrence of a pancreatic fistula and patient death, as previously reported [12,56].

Currently, the definition of the pancreatic fistula and grading is based on the updated International Study Group on Pancreatic fistula (ISGPF) postoperative pancreatic fistula grading from 2016. The ISGPF defines the pancreatic fistula based on amylase activity in the drained volume that is increased by more than three times compared with the upper normal serum level; the definition establishes no minimum restrictions in the drained fluid volume output to define pancreatic fistula [34]. Therefore, according to the ISGPF definitions, the amylase activity level in the drained fluid on the first postoperative day is a major predictor and indicator of pancreatic fistula occurrence. In the ISGPF grading, a grade A fistula is no longer regarded as a true, clinically significant fistula and considered only a “biochemical fistula”—if no drains are placed in the patient, it is not recorded [34,52]. In this scenario, no particular therapeutic care is required. Conversely, a grade B fistula is a real, clinically relevant fistula that is associated with a high risk of aggravation towards a grade C fistula by the development of organ failure and systemic complications (myocardial infarction, thromboembolism, renal failure). It requires specific therapeutic management, such as maintenance of the drains for 3 weeks after the operation, with the patients being frequently discharged with the drains in place; image-guided percutaneous or endoscopic drainage of intra-abdominal collections or interventional repositioning of the drains guided by imagistic methods; nothing by mouth with parenteral nutrition and enteral nutritional support; antibiotics to control the infection; and somatostatin analogues [18,34]. A grade C fistula is life-threatening as it is associated with organ failure (cardiac, respiratory, renal, etc.), clinical instability, and frequent patient death. It requires intensive care support (intubation, hemodialysis, inotropic agents) and surgical reinterventions after the failure of percutaneous/endoscopic drainage. However, often, surgical reinterventions for fistula treatment fail to improve the prognosis of the patient [18,34,36,57,58].

In this context, understanding the therapeutically controllable factors that are associated with the occurrence of pancreatic fistulas becomes of primary importance. Several pre- and postoperative predictors for the occurrence of postoperative pancreatic fistulas have been reported by various studies until now, such as tumor-related factors (the type of pancreatic tumor, histopathologic subtype, its dimension), patient-related factors (age, sex, obesity, fat distribution, diabetes mellitus, cardiovascular comorbidities), pancreas-related factors (texture of the pancreas—hard versus soft, diameter of the main pancreatic duct), operative-related factors (type of pancreatic anastomosis, use of intrapancreatic stents or not, degree of intraoperative hemorrhage, operative time), postoperative factors (occurrence of complications, the precocity of their diagnosis, and their management), and the surgeon’s expertise [36,40]. Our study has revealed the following significant predictive factors for the occurrence of a postoperative pancreatic fistula: patient-related factors such as preoperative weight loss and hypoalbuminemia; pancreatic-related factors such as soft pancreatic texture and a non-dilated main pancreatic duct; operative-related factors (the occurrence of important intraoperative hemorrhage); and postoperative factors (the occurrence of postoperative complications). The soft consistency of the pancreas has been reported by many authors to be a risk factor for the occurrence of postoperative pancreatic leakage (even the most important predictor) and other complications, becoming a proposed intraoperative criterion for the choice between a pancreaticogastrostomy and a pancreaticojejunostomy [1,8,17,19,41,58]. Therefore, to circumvent such a negative predictive factor, a preoperative octreotide to increase the consistency of the pancreas was proposed [8]. A soft pancreatic consistency is usually associated with ampullary or distal bile duct tumors, cystic pancreatic cancer, or neuroendocrine tumors; as it is correlated with a higher risk of injury and ischemia, and suggests a preserved exocrine pancreatic function, it is a strong predictor for the risk of exposure of the anastomosis to the digestive enzymes and occurrence of the fistula [8,9]. Instead, a hard consistency is associated with chronic pancreatitis, fibrosis, and pancreatic duct adenocarcinoma [8]. In such a context, neoadjuvant therapy alongside its putative role in tumor downstaging, by inducing tissue fibrosis, could be a protective factor against postoperative pancreatic fistula occurrence. The pancreatic consistency can be objectively evaluated preoperatively via modern analysis of 3D-CT images, dynamic MRI, or exocrine pancreatic function tests [8,9],; however, it is most frequently achieved intraoperatively, through palpation by an experienced surgeon [8,43] or even by exploiting special instruments such as durometers, as proposed by Kim et al. [28]. The consistency of the pancreas was reported to be of importance not only for the occurrence of postoperative pancreatic fistulas but for postoperative pancreatic insufficiency as well [8,49]. Also, the diameter of the pancreatic duct is a predictor of pancreatic leakage, but it also holds a supplementary significance as well, as a small diameter is associated more frequently with the fibrosis and atrophy of the remaining pancreas and, therefore, with postoperative pancreatic insufficiency [41,49,58]. Further, patient preoperative weight loss has been highlighted by other authors as well, being a significant predictive factor for the occurrence of postoperative pancreatic leakage or even the only significant predictor [8,9]. Intraoperative blood loss has also been found by other authors to be a predictor for fistula occurrence, although the exact explanation (hypoperfusion/ischemia or edema due to excessive fluid resuscitation) behind such an association is insufficiently understood [9]. In this regard, a surgical artery first approach to prevent the intraoperative excessive blood loss (such as a right posterior approach pancreaticoduodenectomy or other variant) or even a laparoscopic approach could be recommended [5,51,59,60].

The predictive factors revealed by our statistical analysis have been reported by other authors as well, being also included in the currently existing pancreatic fistula scores [8,9,35,36,39,43,61]. However, there is an inconsistency between various studies regarding other predictors of pancreatic fistula occurrence [2]. In this concern, some of the putative predictors are considered significant by almost all the authors and are included in the fistula scores (e.g., pancreas texture, pancreatic diameter). However, some authors consider other predictors as well, usually within a range of 2 to 5 predictors. For example, patient obesity, fat distribution, smoking status, some comorbidities, age or sex, leukocyte count, C reactive protein (CRP) value, and heart rate are considered predictors by some authors, while other studies did not validate them as being of relevance [36]. In this context, we could not find a statistically significant association between the occurrence of a pancreatic fistula and several putative predictors reported by other authors, such as patient comorbidities—obesity, diabetes mellitus, cardiovascular comorbidities, age, sex, and smoker status; tumor-related factors—histopathologic type, origin (ampullary, duodenal, cystic), and dimension; operative factors—intrapancreatic duct stenting; and type of pancreatic anastomosis—pancreaticogastrostomy versus pancreaticojejunostomy. In this concern, the study of Bhoriwal et al. from 2021 on 35 patients regarding the comparable outcomes between the use of internal stents and unstented duct-to-mucosa pancreaticogastrostomy [3] is in consensus with our statistical finding, while other authors consider internal stents safer [2]. However, we did find a positive significant correlation between patient obesity and soft pancreatic consistency, which is a predictor of postoperative pancreatic fistulas, as reported by other authors as well [8]. It is interesting to notice the inconsistent findings/lack of statistical significance for individual risk factors of pancreatic tumor nature, histopathologic type, and dimension, and several patient characteristics findings (age, sex, patient comorbidities including cardiovascular disease, diabetes mellitus, obesity) for the risk of fistula development and patient prognosis are similar to the reports of other authors as well [2,8]. At the same time, such findings regarding pancreatic/periampullary tumors are similar to the reports on other types of retroperitoneal tumors, such as primary retroperitoneal tumors [62,63].

Several factors have been included in the existing pancreatic fistula scores that help in predicting the risk of pancreatic fistula occurrence after surgery, such as the pancreatic gland texture, pancreatic diameter, intraoperative blood loss, histopathologic type of the pancreatic/periampullary lesion, and others [8,9,35,61]. In this regard, several pancreatic fistula scores have been proposed, such as Callery, Wellner, Roberts, Yamamoto, and even online fistula score calculators [9,35,36,37,61]. Of the proposed scores, the Callery score from 2013 appears to be the most appreciated, as several studies have found a good positive correlation between it and the actual occurrence of the pancreatic fistula, while the Yamamoto score appears to be the most critiqued, as no predictive significance was reported when tested in a few studies. The Callery score has three models: model I, where one point is allocated for each pancreatic fistula risk factor; model II, in which the magnitude of the beta-coefficients from the regression equation is also included in the score; and model III, which is similar to model II but is easier to use as it has been simplified. The Callery score considers the following pancreatic fistula risk factors: pancreas texture (firm versus soft), the origin and histopathologic type of the tumor (ampullary, duodenal, cystic, pancreatic islet cell tumor; pancreatic adenocarcinoma, pancreatitis), the diameter of the main pancreatic duct (less than 1 mm; 2 mm; 3 mm; 4 mm; 5 mm or more), and the severity of intraoperative blood loss (with the following categories: less than or equal to 400 mL; between 401 and 700 mL; and between 701 and 1000 mL). Based on such risk factors, the Callery score considers four groups of risk for the occurrence of the pancreatic fistula (negligible, low, intermediate, and high risk) and proposes a differentiated therapeutic attitude based on the patient risk score [9].

However, even if the Callery score appears more suitable in predicting pancreatic fistula risk, it is not regarded as ideal. There is still no unanimously approved fistula score, and efforts are being made to elaborate a superior score. Nonetheless, the validation of an improved fistula score would be essential for more adequate therapeutic management of the patients. Such a score could enable a better selection of operable patients; an improved preoperative treatment of the cases, with the correction of key biological parameters; a better intraoperative approach, with the choice of the best surgical approach to control blood loss and the most adequate pancreatic anastomosis type to decrease the risk of the postoperative fistula; and an early diagnosis of the pancreatic fistula based on the calculated/anticipated patient individual risk and, therefore, a more adequate treatment of the pancreatic fistulas based on the patient individual risk score, as highlighted by other authors as well [9,36,62].

In such a context, we designed and tested a postoperative pancreatic fistula score that we consider useful as it is based only on predictive factors that have been validated by the majority of previous studies. We have allocated one point to each of the following risk factors for the occurrence of pancreatic fistulas: soft pancreas, non-dilated Wirsung, intraoperative blood loss, perioperative complications, preoperative patient hypoalbuminemia, and weight loss. Based on these factors, we generated a 0–6 point pancreatic fistula score.

Following statistical testing of the elaborated score, we considered the following degrees of fistula risk: unlikely fistula—0 points; low risk of fistula—between 1 and 3 points; and high risk of pancreatic fistula—between 4 and 6 risk points. Such a pancreatic fistula score has led to improved patient risk stratification, as it associated a significant difference between the three risk score groups and the postoperative occurrence of the pancreatic fistulas. Our study has also found a significant positive association between the pancreatic fistula risk score proposed by us and patient death. We consider that such a pancreatic score, as proposed by us, would be extremely useful in predicting the pancreatic fistula risk as it is based on predictors that have been consensually reported by authors across time but it is also simple and easy to use. It also helps in directing improved management of the patients, from the selection of the operable patients and their preoperative preparation to the choice of the operative technique and postoperative care. In this regard, our study has revealed another important finding: the choice of pancreatic anastomosis should also be based on fistula risk because, in high-risk patients, undertaking a pancreaticogastrostomy is associated with a significantly lower risk of postoperative pancreatic fistulas than that of a pancreaticojejunostomy. We consider such a finding to be of relevance, as currently there is still no consensus between the authors on the best anastomotic technique, with both the pancreaticojejunostomy and pancreaticogastrostomy procedures being extremely technically challenging and having associated advantages and disadvantages. However, another key factor to be considered is the training, expertise, and preference of the surgeon based on the intraoperative anatomic findings [1]. Another limiting factor is in the existing technical facilities of the hospital/clinic, with the best results being achieved in high-volume, dedicated medical centers [7,19,23,40,50].

Therefore, the design of an improved fistula risk prediction score would enable more adequate, personalized therapeutic management of the patients based on their particular fistula risk. For example, in high-risk patients, the following could apply: the choice of a pancreaticogastrostomy, which was not proven to diminish mortality but could decrease the morbidity rate (rates of pancreatic fistula); a posterior surgical approach; the use of external pancreatic drains; somatostatin analogues, such as pasireotide (although with contradictory results, beneficial vs. no effects); anastomotic sealants; and delayed abdominal drain removal. Instead, in low-risk patients, the surgeon could consider the possibility of not placing intra-abdominal drains and of early patient feeding; also, such cases could be suitable for the training of surgical residents [64,65,66].

Limitations of the study

Our study has several limitations: the unicentric, low-volume, and retrospective character of the study; lack of randomization (the surgeon has intraoperatively chosen the type of pancreatic anastomosis based on the local anatomic findings); and changes and improvements in the diagnostic and surgical techniques, perioperative care, definitions and grading of fistula, the gain of surgical experience that occurred with time, and in the continuous changing medical landscape. However, the fistula risk score proposed in the current study is based on predictors that have been consensually found by other previous studies as well. Also, we aimed to present our experience regarding the occurrence of pancreatic fistulas after cephalic pancreatectomy, to propose a model of fistula risk score to be verified by larger studies and to raise a new question: if the differences between pancreaticogastrostomy and pancreaticojejunostomy should not be looked for except in the case of patients with a high risk of fistula (targeted search).

## 5. Conclusions

Despite the decrease in the mortality rates following cephalic pancreaticoduodenectomy, the morbidity rates are still high, associated with a significant percentage of postoperative pancreatic fistulas. Therefore, knowledge of the therapeutically controllable factors that could decrease the rate of pancreatic fistulas and other postoperative complications is essential to increase the overall survival of the patients. Currently, there is no consensus regarding the ideal surgical anastomosis type, and the question of the choice between pancreaticogastrostomy and pancreaticojejunostomy considered by authors is still open. Also, currently, the worldwide implementation of an ideal pancreatic fistula risk score is missing. As the pancreatic fistula risk scores already proposed by various authors have not been consensually validated (not even the Callery score, which is the most cited), a simpler risk score based on significant predictors should be designed, tested, and implemented.

A simpler pancreatic fistula risk score, based on significant predictors for the occurrence of postoperative pancreatic fistulas, is essential for the differentiated intraoperative approach of each case—including the choice of the surgical technique/anastomosis—for the early fistula detection and efficient therapeutic management of each patient based on individual risk scores.

Our study has revealed several predictive factors for the postoperative occurrence of fistulas that have been validated by previously published studies as well: the soft consistency of the pancreas, non-dilated Wirsung, important intraoperative blood loss, other perioperative complications, preoperative patient hypoalbuminemia, and patient weight loss. The occurrence of pancreatic fistulas has been significantly associated with the development of other postoperative major complications and patient death.

Similar to other studies, when analyzing the entire group of patients (low- as well as high-risk patients), no significant differences in the pancreatic fistula rate could be found between the patients where a pancreaticogastrostomy was performed compared with those where a pancreaticojejunostomy was conducted. Interestingly, however, when analyzing only the high-risk group of patients, our study has revealed a supplementary finding: for the association of patients’ fistula risk factors, the pancreaticogastrostomy was significantly associated with a lower pancreatic fistula rate than the pancreaticojejunostomy. Therefore, if our findings were validated by larger studies, pancreaticogastrostomy could be regarded as safer and considered preferentially when dealing with high-risk patients. We consider that a pancreaticogastrostomy could be safer than a pancreaticojejunostomy in pancreatic fistula high-risk patients (with satisfactory results according to our study), taking into consideration the superior plasticity of the structures, improved healing, and better vascularization of the gastric wall versus the jejunal wall, despite the higher rates of hemorrhage that may be associated with such an anastomosis.

Based on fistula predictors reported both by other authors and our study, to decrease the risk of postoperative pancreatic fistulas, several factors should be considered: a judicious selection of the operable patients; the achievement of good preoperative health status for all the patients, with the correction of their disequilibria (hypoalbuminemia, underweight status, excessive weight—correlated with a soft pancreas); an adequate intraoperative, technical, instrumental endowment; and the adaptation of the surgical approach and technique based on the individual risk of each patient. As the pancreatic consistency, diameter of the Wirsung, and proximity/involvement of the blood vessels appear to be essential for the surgical approach and success of the anastomosis, thorough knowledge of these factors should be obtained via CT imaging with 3D reconstructions and density analysis, implementation of durometers, and adequate preparation for vascular surgery. In such a context, better imagistic and surgical instrumentation should be implemented for this extremely complex type of surgery. However, despite these factors, a major determinant should always remain as the experience of the surgeon with a particular technique and preference based on the intraoperative anatomic findings. In such a context, complex, high-risk patients should be preferably treated in high-volume, dedicated centers.

Our study, similar to others, has several limitations: a small group of patients; unicentric and retrospective nature with putative lack of data; lack of randomization; changes in the definition and grading, diagnostics, surgical techniques; and improvements in perioperative care that have occurred during the study period. Such limitations form the complexity and beauty of research but also raise difficulties in making easy conclusions. Nonetheless, our study has revealed similar predictors as those reported by previous large-scale studies, therefore proving to be of significance. We have also raised a new question: whether the differences between the results of pancreaticogastrostomy versus pancreaticojejunostomy should be searched not only in the high-risk patient groups. Therefore, despite its limitations, the proposed pancreatic fistula risk score based on major fistula predictors could contribute to the integrative view of the results of multiple centers and to the generation of new concepts/frames of analysis to elaborate on the improved management of cephalic pancreaticoduodenectomy patients. In this regard, the validation of our fistula risk score can come only from future extended, large-scale, worldwide, multicentric studies.

## Figures and Tables

**Table 1:** Histopathologic diagnostic for the patients where cephalic pancreaticoduodenectomy was performed.

Histopathologic Diagnostic	Number of Cases	Percent of Cases
Pancreatic adenocarcinoma	51	48.57
Ampullary adenocarcinoma	16	15.24
Neoplastic pancreatic cysts	4	3.81
Cholangiocarcinoma	15	14.29
Duodenal carcinoma	14	13.33
Chronic pancreatitis	2	1.90
Other	3	2.86

**Table 2:** The proposal of a new pancreatic fistula score.

Risk of Postoperative Pancreatic Fistula	Number of Risk Points
fistula unlikely	0 points
low risk of fistula	1–2−3 points
high risk of fistula	4–6 points

## Data Availability

No new data were created or analyzed in this study.
